# Trigeminal Neuropathy Ascribed to a Probable Intrinsic Brainstem Schwannoma of the Pons: A Case Report

**DOI:** 10.7759/cureus.18764

**Published:** 2021-10-14

**Authors:** Hani Chanbour, Ahmad Jiblawi, Azzam Taybah, Jad El Masri, Khaled Jiblawi

**Affiliations:** 1 Medicine, Faculty of Medical Sciences - Lebanese University, Beirut, LBN; 2 Radiology, American University of Beirut Medical Center, Beirut, LBN; 3 Radiology, Beirut Arab University, Beirut, LBN; 4 Neurosciences, Faculty of Medical Sciences - Lebanese University, Beirut, LBN

**Keywords:** tumor, intraparenchymal, pons, brainstem, trigeminal neuralgia, schwannoma

## Abstract

Brainstem schwannomas are very rare, only 11 cases have been reported in the literature so far. We report a small intraparenchymal brainstem schwannoma of the pons, in a 37-year-old female patient who presented with a four-day history of numbness at the mandibular division of the trigeminal nerve on the left side of her face. Trigeminal neuralgia was diagnosed, and magnetic resonance imaging (MRI) showed a small intraparenchymal lesion at the level of the nuclei of the left trigeminal nerve present at the junction between the pons and left brachium pontis. A biopsy wasn’t feasible in this small lesion. We discuss the keen radiological features that helped in the presumptive diagnosis of an intrinsic brainstem schwannoma, with both intra- and extra-axial components. Our case had the earliest presentation and the smallest probable brainstem schwannoma reported so far, as well as its unique symptomatology of trigeminal neuralgia related to both the nucleus and the nerve of the fifth cranial nerve (CN V).

## Introduction

Schwannomas are tumors derived from the schwann cells that form the myelin sheath of the peripheral nerves [1]. They account for 8% of intracranial tumors [1,2] with the majority emerging from the acoustic and trigeminal nerves in the cerebellopontine angle [1].

Intra-axial schwannomas are relatively rare. Less than 1% of schwannomas occur within the brain parenchyma [1,3], and only 85 other cases have been reported so far [4]. The most common sites are the frontal and temporal lobes, the vermis, and the lateral and fourth ventricles [1,2,5]. The first case involving the brainstem was described by Prakash B et al. in 1980 [6]. Since then, less than 10 cases of intraparenchymal schwannomas located in the brain stem were reported in the literature [1,7].

We present the case of a 37-year-old female patient who harbored a probable intraparenchymal schwannoma of the pons and describe its neuroradiological characteristics. In addition, we briefly review the main theories of the origin of brainstem schwannomas, along with its common radiological features.

## Case presentation

 

A 37-year-old woman previously healthy presented with four days history of numbness at the mandibular division (V3) of the trigeminal nerve on the left side of her face. Physical exam revealed an alert, calm well-oriented patient and demonstrated that binocular vision was normal, bilateral pupils were of the same size, eyes were sensitive to light reflection and eyeballs moved freely in all directions. The fundoscopic exam was normal and facial sensation was also normal except for numbness and sensory loss at the mandibular division of the trigeminal nerve CN V on the left side of her face. The findings were solely unilateral. There was no nystagmus, no mouth deviation, and no sensorineural hearing problems. Otherwise, cranial nerve examination was normal. No cutaneous stigmata of neurofibromatosis type 1 were observed. She was diagnosed with trigeminal neuropathy and magnetic resonance imaging (MRI) of the brain was ordered.

**Figure 1:**
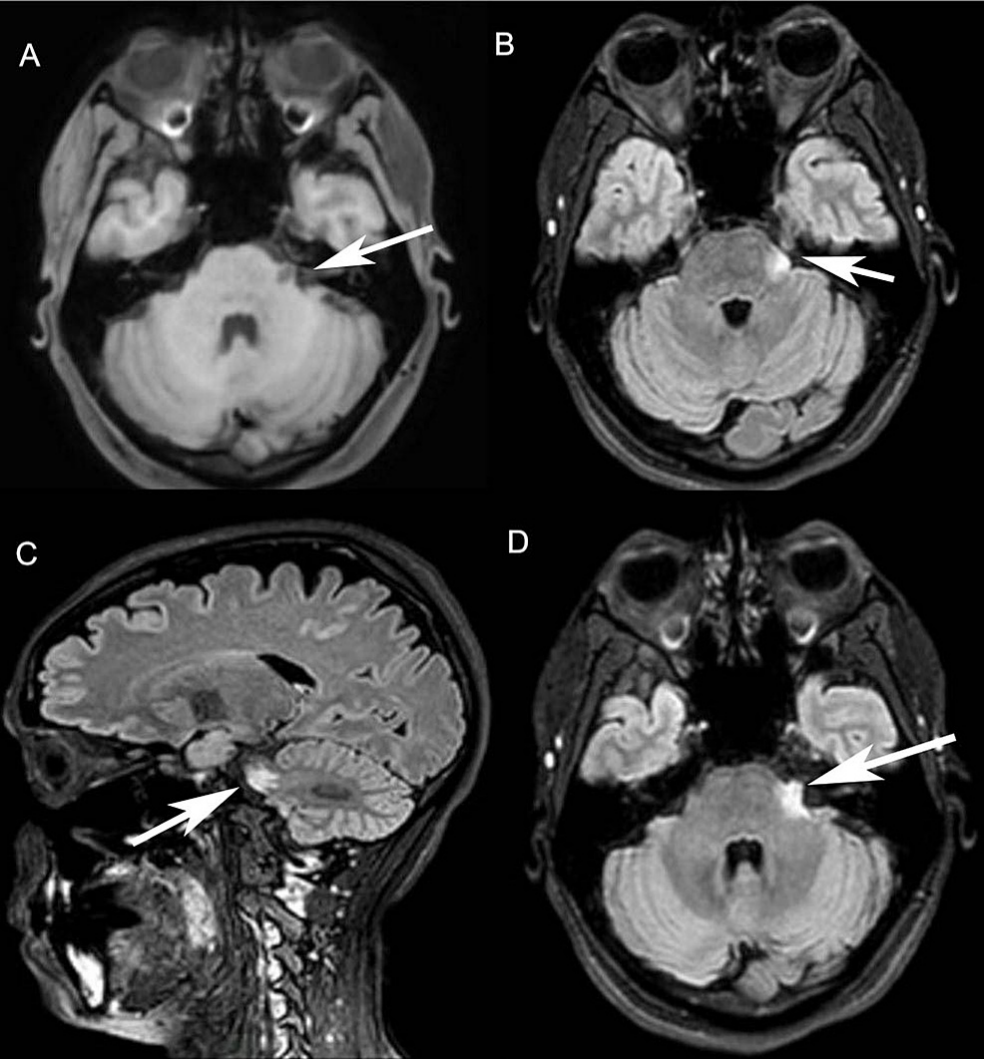
(A) T1-weighted image: Axial plane showing a hypo-intense small intraparenchymal lesion measuring 7 mm in anteroposterior diameter at the level of the nuclei of the left trigeminal nerve present at the junction between the pons and left brachium pontis. (B) FLAIR weighted sequence: Axial plane showing a wider hyper-intense intraparenchymal lesion measuring 14 mm in anteroposterior diameter and 7 mm in thickness at the level of the nuclei of the left trigeminal nerve present at the junction between the pons and left brachium pontis, appearing larger than its T1 counterpart. (C) FLAIR weighted sequence: Sagittal plane showing the hyper-intense pontine intraparenchymal lesion measuring 9 mm in superoinferior diameter. (D) FLAIR weighted sequence: Axial plane showing a hyper-intense small extraparenchymal lesion at the level of the nuclei of the left trigeminal nerve at the junction between the pons and left brachium pontis, measuring 4 mm extending along the left trigeminal nerve that is thickened in its preganglionic portion before the left Meckel’s cave in addition to the intraparenchymal lesion. FLAIR: Fluid-attenuated inversion recovery

The MRI of the brain was performed on a 3T Ingenia Philips (Eindhoven, The Netherlands). It showed a small intraparenchymal lesion at the level of the nuclei of the left trigeminal nerve at the junction between the pons and left brachium pontis with low signal in T1 (Figure 1 A) as well as T2 signal abnormality. It showed a wider high signal on fluid-attenuated inversion recovery (FLAIR) sequence, measuring 14 mm in anteroposterior diameter 7 mm in thickness (Figure 1 B) and 9 mm in superoinferior diameter (Figure 1 C), with a small extraparenchymal extension along the left trigeminal nerve which is thickened in its pre-ganglionic portion before the left Meckel’s cave. The intraparenchymal enhancing core is estimated at around 7 mm with a small 4 mm extraparenchymal enhancing extension (Figure 1 D), with both showing faint diffusion restriction. Both the left VII and VIII cranial nerves complex were of normal outline, thickness, and signal throughout their course into the left internal auditory canal, without abnormal enhancement or associated lesion defined.

**Figure 2:**
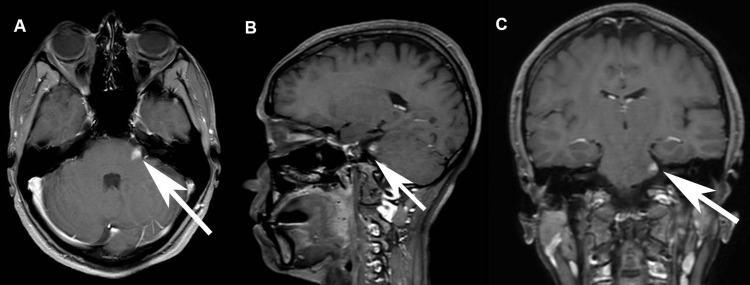
Avid enhancement of small lesion at the origin of the left fifth cranial nerve being partly extra-axial and partly intra-axial in the pons. A: T1W axial MRI brain with gadolinium enhancement B: T1W sagittal MRI brain with gadolinium enhancement C: T1W coronal MRI brain with gadolinium enhancement

The T1-weighted MRI images showing enhanced brainstem lesions are presented in Figure 2.

Based on the radiological findings, a presumptive diagnosis of brainstem schwannoma was most probable. The tumor was not amenable to biopsy due to its very small size, serial follow-ups are considered to implement the relevant management.

## Discussion

Brainstem schwannomas are a rare occurrence. To our knowledge, there are 11 cases of brainstem schwannomas reported in the literature so far. We herein describe the case of a probable brainstem schwannoma of the pons, presenting as trigeminal neuropathy. Intraparenchymal schwannomas are extremely rare lesions. In comparison with vestibular schwannomas, they usually occur during the second and third decades [1], as in our case.

It is difficult to explain the origin of intracranial parenchymal schwannomas since it is known that brain parenchyma normally lacks the presence of schwann cells [3]. Several authors proposed myriad hypotheses, with the most accepted one suggesting that the origin of intraparenchymal schwannomas would be schwann cells around arteries in the intracranial perivascular nerve plexuses in the brain and the subarachnoid space [1,2,3,8]. Alternative theories involve the growing of neural crest cells trapped during embryogenesis [8], differentiation of pluripotential mesodermal pial cells [1,8,9] schwannosis, or aberrant schwan cells having neoplastic potential, and schwann cell migration through the trigeminal nerve [8]. Schwann cells were also reported in brain tissue in multiple sclerosis plaques or at the edge of old infarcts [9].

Generally, characteristic imaging findings for a schwannoma include solid component appearing as hypo-intensity and hyper-intensity on T1 and T2 imaging [1,5,6] with the lesion being well-circumscribed typically with calcification, peritumoral edema [3], hyper-intensity of the surrounding brain in the FLAIR sequence [1], gliosis and superficial or periventricular location [1,2]. However, in the case of intraparenchymal schwannomas, it is important to note that there are no pathognomonic features on neuroimaging [1].

The differential diagnosis of intraparenchymal schwannoma might include hemangiopericytoma, meningioma, pleomorphic xanthoastrocytoma, dysembryoplastic neuroepithelial tumor (DNET), or ganglioglioma [1,2]. Usually, it is difficult to differentiate between an intra-axial schwannoma and a glioma by using MRI [2,5], particularly in the absence of cranial nerve involvement [3]. This wasn't valid for our current case, which had the involvement of the trigeminal nerve apparent both clinically and radiologically. In addition, our patient’s MRI shows an extra-axial extension component along with the left trigeminal nerve manifested as thickening in its left preganglionic portion before the left Meckel's cave.

Our case has unique and interesting features compared with other cases. One, the symptoms in our patient developed four days before presentation; in other described cases, brainstem schwannomas symptoms developed between two weeks to three years [1,7], thus making our patient the earliest presentation being documented on imaging. Two, our patient has the smallest measured documented lesion. Three, the described lesion has both an intraparenchymal and an extraparenchymal component. Four, this case is the second to have atypical features, including the absence of cystic component, calcification, or vascularization [3]. Five, our case is the sixth one with recorded T2 signal abnormality.

## Conclusions

Intrinsic brainstem schwannomas are exceptionally rare. Preoperative radiological diagnosis is exceedingly difficult since there is no clear consensus on neuroimaging features of schwannomas. Our unique case of pons schwannoma involving the trigeminal nucleus and nerve has the earliest presentation, the smallest size, and both intra and extraparenchymal components.
